# Observation of a Flowing Duct in the Abdominal Wall by Using Nanoparticles

**DOI:** 10.1371/journal.pone.0150423

**Published:** 2016-03-03

**Authors:** HyunSuk Jang, Joohwan Yoon, HyunJi Gil, Sharon Jiyoon Jung, Min-Suk Kim, Jin-Kyu Lee, Young-Jae Kim, Kwang-Sup Soh

**Affiliations:** 1 Nano Primo Research Center, Advanced Institute of Convergence Technology, Seoul National University, Suwon, 443–270, Korea; 2 College of Physical Education, University of Suwon, Hwaseong, 445–743, Korea; 3 Department of Medical Engineering, Konyang Univiersity, Nonsan-si, 320–711, Korea; 4 Department of Chemistry, Seoul National University, Seoul, 151–747, Korea; Hanyang University, REPUBLIC OF KOREA

## Abstract

The primo vascular system (PVS) is being established as a circulatory system that corresponds to acupuncture meridians. There have been two critical questions in making the PVS accepted as a novel liquid flowing system. The first one was directly to show the flow of liquid in PVS and the second one was to explain why it was not observed in the conventional histological study of animal tissues. Flow in the PVS in the abdominal cavity was previously verified by injecting Alcian blue into a primo node. However, the tracing of the dye to other subsystems of the PVS has not been done. In the current work we injected fluorescent nanoparticles (FNPs) into a primo node and traced them along a primo vessel which was inside a fat tissue in the abdominal wall. Linea alba is a white middle line in the abdominal skin of a mammal and a band of fat tissue is located in parallel to the linea alba in the parietal side of the abdominal wall of a rat. In this fat band a primo vessel runs parallel to the prominent blood vessels in the fat band and is located just inside the parietal peritoneum. About the second question on the reason why the PVS was not in conventional histological study the current work provided the answer. Histological analysis with hematoxyline and eosine, Masson’s trichrome, and Toluidine blue could not discriminate the primo vessel even when we knew the location of the PVS by the trace of the FNPs. This clearly explains why the PVS is hard to observe in conventional histology: it is not a matter of resolution but the contrast. The PVS has very similar structure to the connective tissues that surround the PVS. In the current work we propose a method to find the PVS: Observation of mast cell distribution with toluidine blue staining and the PN has a high density of mast cells, while the lymph node has low density.

## 1. Introduction

The primo vascular system (PVS) was proposed to be a circulatory system corresponding to acupuncture meridians of humans and mammals by BH Kim [1]. The extensive network of the PVS which is a system composed of the primo vessels (PVs) primo nodes (PNs) and fluid that flows in the PVS was confirmed in various organs of mice, rats, rabbits, dogs, pigs, cows, and humans [2, 3]. Most of the experiments were done with vivi-staining of the PVS for its visualization using dyes such as Trypan blue, Alcian blue, chrome-hematoxylin and hemacolor [4].

BH Kim’s team reportedly used some blue dye to trace the acupuncture meridians and found the PVS network unexpectedly [1], but the method was not fully presented so that other independent groups can reproduce their results. Until the present time no one has been able to trace the acupuncture meridians by this type of method. The current work reports an interim progress in tracing the PVS which are near the conception vessel (CV) of a rat by injecting fluorescent nanoparticles (FNP) into a PN.

**Fig 1:**
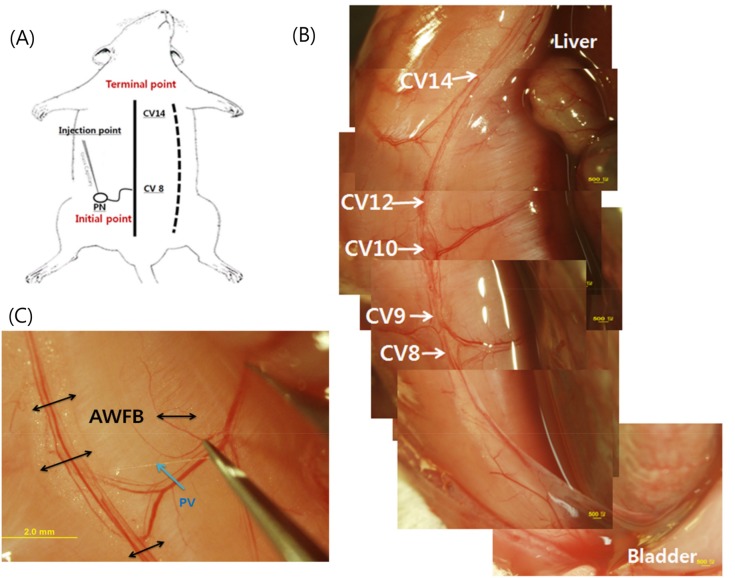
The anatomical position of the novel flowing duct in the abdominal wall fat band. A: Schematic illustration showing the location of linea alba and the conception vessel CV in the abdominal skin side. The broken line is the surgery cutting line of the abdominal wall. The line is in the right hand side from the linea alba in order to avoid cutting the PVS in the AWFB. The FNPs that were injected to a PN entered the AWFB and appeared at the terminal point to be continued to the PVS on the liver surface. B: The blood vessels in the AWFB inside the parietal peritoneum of the abdominal wall. The locations CV 8 to 14 are mere markings for positional references and not real CV-acupoints. Note that the CV8 corresponds to the umbilicus and the parietal peritoneum continues down to the ligament wrapping the bladder. C: A PV (arrow) emerged from the AWFB (double arrows) was raised tautly with a forceps.

In acupuncture theory of meridians the conception vessel runs along the linea alba where the acupoints CV 8 and 14 are at the umbilicus and the xiphoid, respectively, as indicated in Fig 1A. The abdominal wall fat band (AWFB) of a rat is located in the peritoneum side of the abdominal wall along the line corresponding to the CV [5]. There are prominent blood vessels in the AWFB running along the CV line. The caudal extension of the fascia becomes a ligament covering the bladder (Fig 1B). The AWFB is indicated with an arrow, and the bulged fat tissue is covered by the peritoneum. A primo vessel (PV) came out of the AWFB (Fig 1C) and joined the organ-surface PVS (OS-PVS).

Even though tracing of the complete path of a PV was not fully done the current work showed two important progresses: The fluid flowed in the PVS when injected into a PN, thereby proving that the PVS was a conduit system [6]; The hard-to-detect PVS surrounded by connective tissues was, for the first time, detected with the aid of flowing rather than staining of a suitable agent.

In addition, the current work produced a significant understanding on why the PVS in skin such as the acupuncture meridians are difficult to observe in conventional histology. We demonstrated that the widely used hematoxylin and eosine (H&E) staining cannot distinguish the PVS from the wrapping connective tissue like the parietal peritoneum. Other staining with Mason’s trichrome or Toluidin blue did not work either. This explains why some histological efforts [7, 8] failed to detect the PVS at acupoints and meridians. The PVS is hard to recognize in the cross sections but is rather distinctively recognizable in the longitudinal sections with its characteristic endothelial nuclei distributions [9]. In the current work we propose an effective method to find the PVS in skin: Observation of mast cell distribution with toluidine blue staining which discriminate the PN from the lymph nodes because the latter has low density of primo nodes.

## 2. Materials and Methods

### 2.1 Animal preparation

The study was performed on sixty male Sprague-Dawley rats, 8–9 weeks old, medium weight 280 ± 10 g, which were obtained from DooYeol Biotech (Seoul, Korea). The rats were housed with a 12/12hr, light/dark schedule in a temperature- and humidity-controlled room, and had *ad-libitum* access to food and water. This study was carried out in strict accordance with the recommendations in the Guide for the Care and Use of Laboratory Animals of the National Institutes of Health. The protocol was approved by the Institutional Animal Care and Use Committee (IACUC) of Woojung BSC, Inc. (Approval Number WJIACUC 20140213-1-05) in conjunction with the Advanced Institute of Convergence Technology, Seoul National University. The rats were anesthetized by intramuscularly injecting a regimen consisting of 0.3 ml of Zoletil and 0.1 ml of Rompun. All surgery was performed under deep anesthesia, all efforts were made to minimize suffering, and the rats were sacrificed by over-anesthetizing without any perfusion.

The entire processes of each experiment took two to three hours in deep anesthesia and no post-surgical analgesia were necessary. During the experiments at least one of the researchers were on site and the conditions of the rats were continually observed. Since healthy rats were used there were no unexpected illness or dyeing occurred during the experiments. Our experimental protocol includes that at the end of the experiments and in the case of unexpected serious illness the rats should be euthanized by over- anesthesia.

An incision of the subcutaneous layer of the abdominal skin along the midline, but slightly off the linea alba, was performed with surgical scissors. We avoided cutting the linea alba in order to maintain the AWFB located at the midline of the ventral peritoneal wall because the target PNs are the AWFB (Fig 1B). All procedures of observations and operations were performed under a fluorescence tissue microscope (MVX10, Olympus, Japan). Once the desired PN was found we proceeded to the injection process. In the whole experiments it was important to keep the PN humid by dripping phosphate buffer solution (PBS) or other suitable liquid onto the PN.

### 2.2 Injection and observation of fluorescent nanoparticles

Fluorescent nanoparticles (FNPs) were synthesized by modifying previously reported method. [10] Modified rhodamine B was incorporated in silica nanoparticles by co-condensation with tetraethyl orthosilicate in ethanol with NH_4_OH and water. The average size of FNPs was 50 nm and FNPs had a photo-luminescence maximum at 547 nm with excitation at 450 nm. The surface of FNPs was functionalized with 2-[methoxy(polyethyleneoxy)propyl] trimethoxysilane (PEG-silane) in order to increase biocompatibility. The PEG modified FNP was dispersed in deionized water (10 mg of FNPs in 1 mL of deionized water).

The FNPs were injected into a PN by a glass capillary which was made sharp by pulling with the glass capillary puller (Narishige, PP830; Tokyo, Japan). In order to minimize the puncture of the PN the tip of the capillary was further sharpened with the Micro-Forge (Narishige, MF-900; Tokyo, Japan). The injecting system was controlled by a home-made 3D manipulator. The glass capillary were connected to a 1ml hypodermic syringe which was supplied with the FNPs through the micro injector (KDS Scientific, KDS Legato 110, Daejon, Korea). The fluid injection was done up to one hour with flow speed 500nl/min.

The flow path of the FNPs was observed with a fluorescent tissue microscope (MVX 10, Olympus, Japan) *in vivo in situ*. In order to study the detailed histological nature of the path the AWFB was harvested and the magnified view of the flow path were recorded with the phase contrast microscope (BX-51, Olympus, Japan)

### 2.3 Histological analysis

The isolated samples were fixed with neutral buffered formalin (NBF) at 23°C for 24 hr ± 2hr. The specimens were embedded in paraffin and cut to 5-μm-thick sections by using a microtome (Reichert Jung 820, Leica, Wetzlar, Germany).

We performed hematoxylin and eosine (H&E), Masson’s trichrome staining following conventional protocols, and picro-sirius red staining (PSR) staining. The protocol by Dolber was adopted for PSR staining [11]. The protocol for the toluidine blue was the same as the one used in [12]. The stained samples were examined under fluorescence microscope (BX-51, Olympus, Japan) with a CCD camera (Infinity 3–1 CCD Camera, Lumenera, USA).

## 3. Results

Fluorescent nanoparticles (FNP) were injected into a PN which was connected to a PV that came out of the AWFB around the point CV 6 near the bladder. Among the sixty rats we found suitable PNs which came out of the AWFB in thirteen rats and the successful injections were made three times. The reason was difficulty with injecting small amount of FNP in a long period over thirty minutes. In the successful cases the FNP flowed in the PV along the CV line in the AWFB which were recorded at two hours after injection. The flow was observed from CV 6 to CV 14 at xiphoid where the PV came out of the AWFB and traced toward the surface of the liver, but further tracing was not possible.

**Fig 2:**
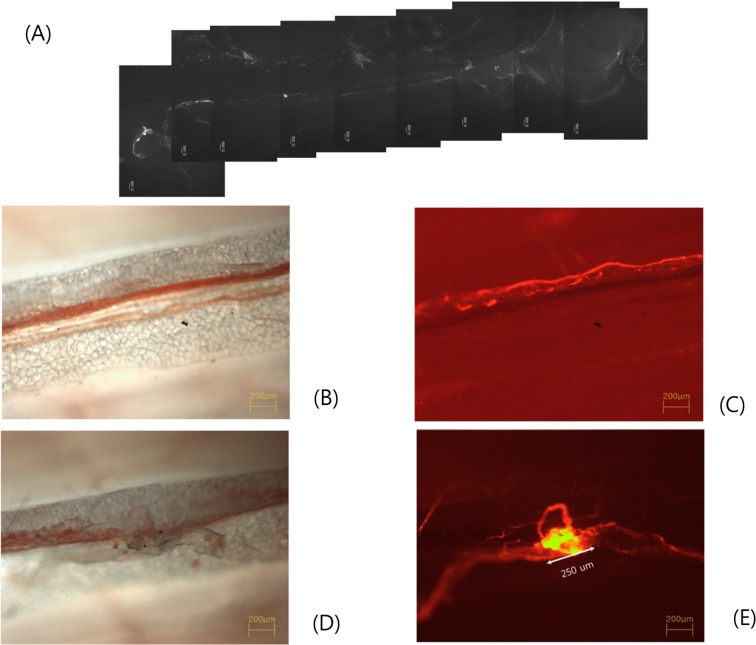
Phase contrast microscope images of the PVS. A: The fluorescent image of the FNPs that were injected at a PN located about the CV4 and flowed in a PVS buried in the adipose tissue of the AWFB. It flowed up to the CV 14 and reemerged to the abdominal cavity toward the liver surface. The flow line was barely visible under the stereo fluorescence microscope. B & C: Phase contrast microscope images of the bright mode (B) and fluorescent mode (C) of the boxed region in (A). The PV (dashed arrow) running parallel to and above the blood vessel (two arrows) is hardly visible in (B) but clearly observable with fluorescence of FNPs in the panel (C). This primo vessel is the first observation of the so called extra vascular PVS. It runs closely along the blood vessel. The AWFB is clearly seen in (B) and its boundary is depicted with two curves in (C). The boundary of the abdominal wall fat band is indicated with two yellow curves. 40x. D & E: The PN is not noticeable without the fluorescence in (D), but manifestly appears with fluorescent view in the panel (E). The size of the PN was 250 μm. The fluorescent nanoparticles were highly concentrated in the PN. 40x

The boxed region of Fig 2A was analyzed with a phase contrast microscope and the magnified view is shown in Fig 2B. Without fluorescen imaging the blood vessel and the fat tissue are easily seen but the primo vessel is difficult to notice. With fluorescence the traces of nanoparticles are clearly manifested thus revealing the PV along the blood vessel (Fig 2C). This is the putative extra-vascular PV along a blood vessel. A PN was observed at a point which was not noticeable without fluorescence (Fig 2D), but its appearance was striking with the fluorescence of nanoparticles (Fig 2E). Its diameter was 250 μm, it was connected to PVs in both sides and a loop of PV was prominent. Similar results of two cases were given as a Supporting Information (S1 Fig).

**Fig 3:**
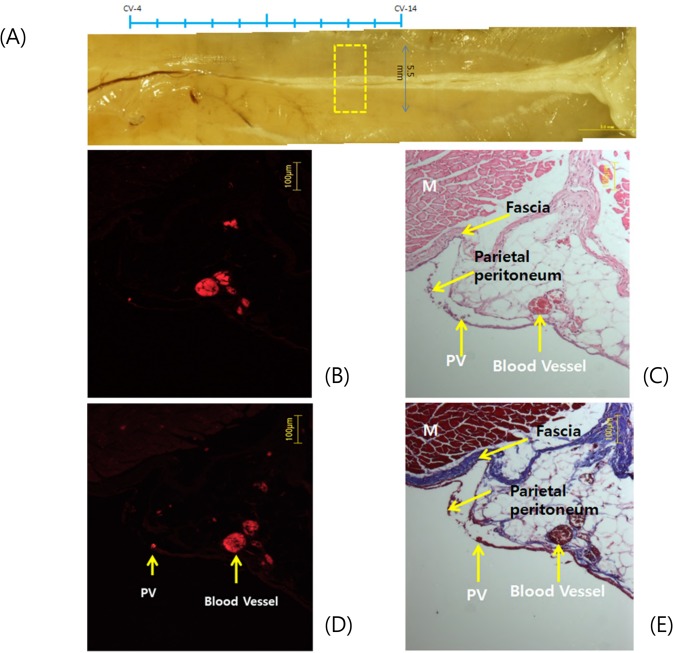
Histological analysis of the PV, peritoneum and fascia. **A:** The position of the tissue block for the cross section. **B:** The spot of fluorescence (arrow) due to FNPs is the position of the PV. Its size is 10 μm and 150 μm away to the left from the large blood vessel. **C:** The H&E staining barely revealed the spot of the PV (arrow) just inside the parietal peritoneum. This figure showed that the PV and the surrounding connective tissue of the parietal peritoneum are not distinguishable with H&E. The deep fascia and the parietal peritoneum are barely distinguishable. Muscle (M) is clearly distinguished by color. B &C are the same sections. **D**: Another section showed the fluorescence spot of the PV (arrow). **E**: The Mason’s trichrome staining cannot distinguish the PV (arrow) and the parietal peritoneum. It can clearly distinguish the parietal peritoneum and the deep fascia. Muscle (M) is also well distinguished. D &E are the same sections.

Fig 3 explains why the PV was not observed in histology with H&E. The fluorescence image revealed the tiny spot of the PV just inside the peritoneum about 150 μm away from the blood vessel (Fig 3C). The region indicated in Fig 3A was analyzed with H&E staining and no noticeable structure was seen (Fig 3B) despite its presence was already proven. In another section the PV was clearly denoted by the fluorescence of nanoparticles that flowed in it (Fig 3D). The Mason’s trichrome discriminates muscle, deep fascia, peritoneum and fat tissue. But the peritoneum and PV could not be distinguished at all (Fig 3E). It is intriguing that the deep fascia and the parietal peritoneum are well distinguished, but the PV is undistinguishable from the surrounding parietal peritoneum.

**Fig 4:**
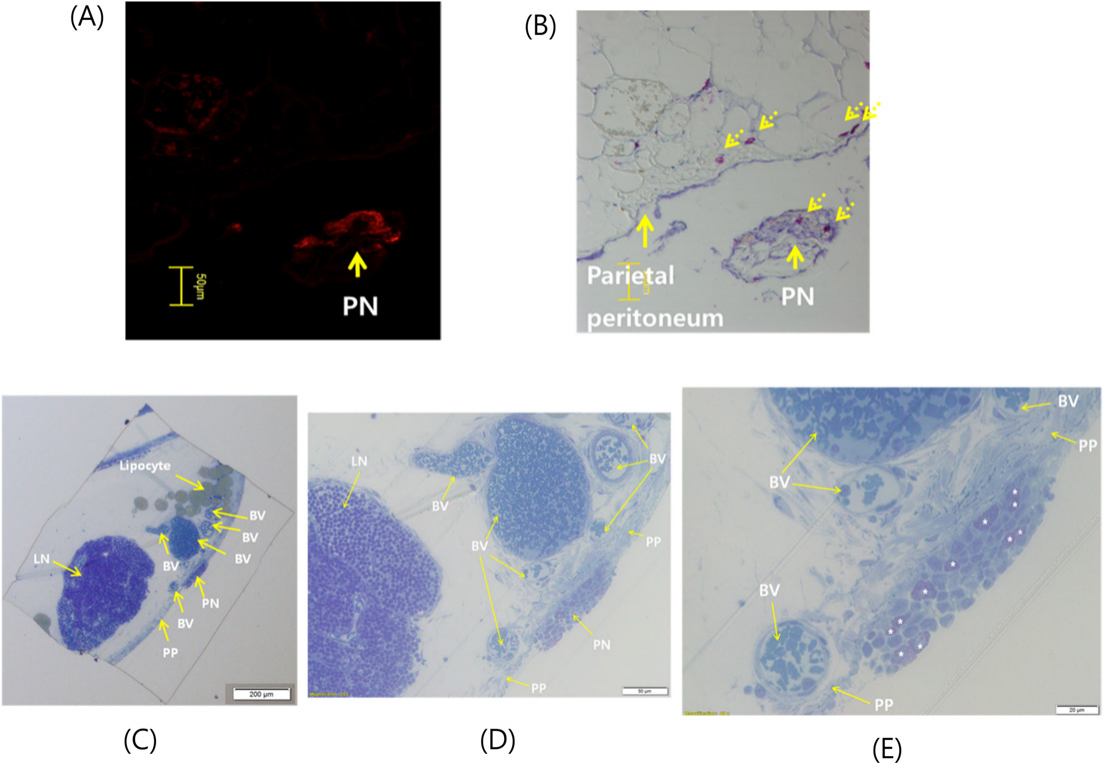
Mast cells in the PN. **A:** The fluorescence of FNPs indicates the location of a PN (arrow). **B**: The toluidine blue staining cannot distinguish the parietal peritoneum and the PN. It showed the presence of mast cells (broken arrows) in the PN. The PN was torn off from the peritoneum during the sectioning process. **C**: An intact PN stained with toluidine blue was obtained. The overview of an AWFB-section shows a PN which are well kept just inside the parietal peritoneum. A lymph node (LN), blood vessels (BV), adipose tissues and parietal peritoneum (PP) are also seen showing their relative locations. It is a rare and fortunate case that a lymph node and a PN located nearby was found as presented in this figure. **D**: A magnified view shows that the cells in the lymph node and the PN look different. In fact, there were no mast cells in the lymph node and but many in the PN. **E**: A further magnified image depicts clearly the distribution of mast cells (*). The toluidine blue staining can be used for identifying the PN by revealing the abundance of mast cells even though it could not distinguish the collagens of the PN and surrounding connective tissues.

The high density of mast cells in the PN [12] was confirmed here again. The presence of the PN was noticed by the fluorescent image of the nanoparticles that flowed in the PN (Fig 4A). In the PN that was inadvertently separated from the deep fascia a mast cell was observed after the toluidine blue staining (Fig 4B). An intact PN was obtained just inside the parietal peritoneum (Fig 4C, 4D and 4E). Even though toluidine blue staining could not show any difference of the PV and the peritoneum it is useful for finding and identifying the PVS by the density of mast cells. In particular it is effective for discriminating lymph nodes and the PN as shown in Fig 4C, 4D and 4E.

## 4. Discussion

The flow of Alcian blue in the PVS was tested in the OS-PVS and the flow speed was measured to be 0.3 mm/sec [6], which was a direct proof that fluid flows in the PVS. In other subsystems of the PVS it was not confirmed whether fluid flows in the PVS. The current study showed that nanoparticles flow in the PVS in the abdominal wall. More importantly, in this study, we found a novel PVS in the middle of the ventral abdominal wall as flowing duct connected to the OS-PVS. It is the first time to show that the PVS can be traced by injecting nanoparticles in a PN. Until the present time the only way to detect some subsystems of the PVS was to visualize them with some staining dyes. The classic work by Kim claimed that the full PVS network was discovered by injecting some blue dye into acupoints of rabbits, but no one could reproduce the results until the present time mainly because the injection methods and procedures were not described in detail [1]. The current work is a significant progress toward tracing of the full PVS network by injecting dyes into a PN.

The limitations of the current tracing experiment are its low repeatability due to the low probability of finding the PN at the abdominal wall. It was necessary to develop a surgery method to avoid cutting the PVS that was present in the AWFB. Therefore the cutting line was on one side off the linear alba. Then it was difficult to evade the blood vessels. In addition, not all rats had the PVS at the expected position. However, through the training of the experimenter the statistics improved better and intact PNs were obtained as shown in Fig 4. Another experimental skill was injecting very slowly the dye into a PN for a long time. Without this the tracing of the PVS for a long distance was not possible. Since the animal was breathing it was extremely difficult to puncture the PN with a micro capillary and keep it in the PN for a long period. Because of these technical difficulties the tracing was only done for a relatively short distance and we could not trace the PVS beyond the AWFB. In order to trace up to the skin and find the acupuncture meridians technical improvements of the injection system is necessary.

The current work added a good insight on why the PVS or acupuncture meridian was not noticed or recognized in numerous conventional histological studies of the skin. As shown in Fig 3 the PV was attached to the parietal peritoneum but its existence was hardly noticeable in the images after staining with H&E, Mason’s trichrome or toluidine blue. This clearly demonstrated the pitfall with conventional histology in searching the PVS.

We found that the Picro-sirius red staining could discriminate the PN from the adjacent peritoneum and deep fascia. The image after the Picro-sirius red staining showed the difference in redness of the three contagious tissues, deep fascia (deep red), parietal peritoneum (dark brown), and the PN (light brown). The section was not in good condition because the peritoneum and the PN were mistakenly separated from the deep fascia, but the distinction between the different tissues was clearly exhibited (Data not shown). However, through our continuing experiments we found that the toluidine blue staining method is more effective and simple for finding and identifying the PN inside connective tissues, as shown in Fig 4 as explained below. In addition, this method clearly distinguishes the PN from lymph nodes, which is of critical importance because there are no simple criteria to distinguish them. Consequently, we propose the researchers to utilize the toluidine method to find the PVS in hitherto unexplored organs like skin.

The high density of mast cells in the acupoints was noticed earlier by acupuncture researchers independent of the PVS teams [13, 14]. The abundance of innate immune cells in the PNs was also repeatedly observed by independent groups [15–18]. In the current work the PN was located in the AWFB which are different from previous organs, and the mast cells were present. This suggests presence of the mast cells as a cytological criterion of the PN. Interestingly, mast cells were rare or absent in lymph nodes. Thus, the immune functions of mast cells in connection with the PVS and lymph system is worthy of further investigation.

## Supporting Information

S1 Fig Flow of Nanoparticles in the PVS. **Fig A in S1 Fig**: The fluorescent image of the FNPs that were injected at a PN located about the CV4 and flowed in a PVS buried in the adipose tissue of the AWFB. It flowed up to the CV 14 and reemerged to the abdominal cavity toward the liver surface. The flow line was barely visible under the stereo fluorescence microscope. **Fig B in S1 Fig**: Phase contrast microscope images of the boxed region in (A). The boundary of the abdominal wall fat band is indicated with two yellow curves. The PN and twisted primo vessel manifestly appear with fluorescent. The size of the PN was 250 μm. The fluorescent nanoparticles were highly concentrated in the PN. 40x. **Fig C in S1 Fig**: Similar to the above case the fluorescent nanoparticles flowed in a primo vessel. **Fig D in S1 Fig**:The PN in which the primo vessel was connected is shown. **Fig E in S1 Fig**: Similarly to the above two cases the flow was traced. **Fig F in S1 Fig**: The PN in which the FNP flowed. **Fig G in S1 Fig**: The primo vessel in which the FNP flowed.
(TIF)
