# Supplementary figures and images for: Observation of a Flowing Duct in the Abdominal Wall by Using Nanoparticles
Source: PLoS One. 2016 Mar 3;11(3):e0150423. doi: 10.1371/journal.pone.0150423 (PMC4777417; doi:10.1371/journal.pone.0150423)

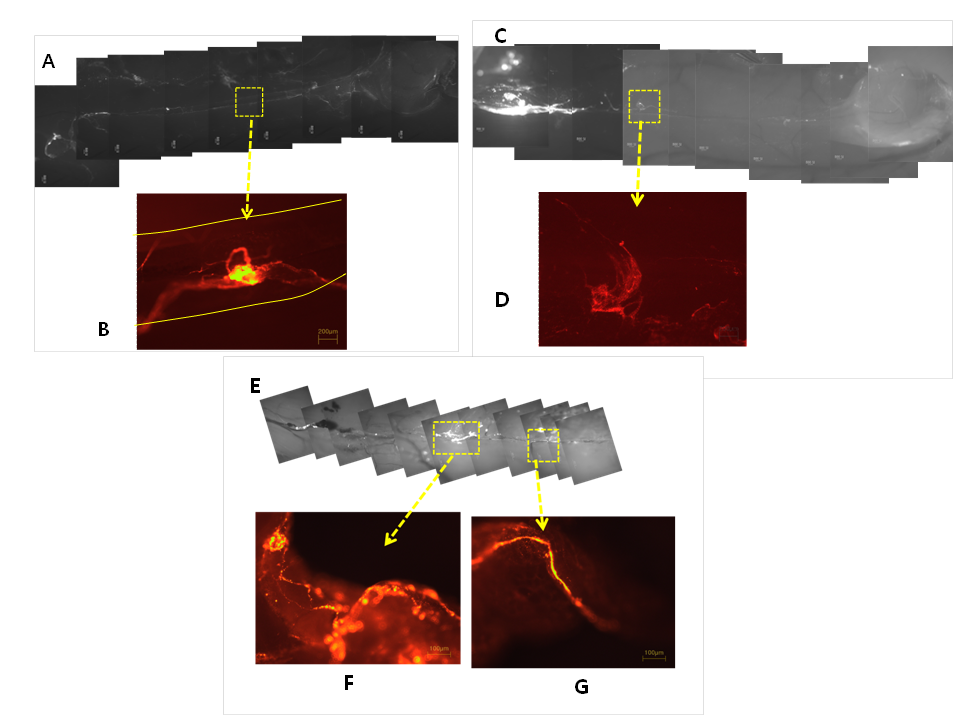

Supplement: S1 Fig — Fig A in S1 Fig: The fluorescent image of the FNPs that were injected at a PN located about the CV4 and flowed in a PVS buried in the adipose tissue of the AWFB. It flowed up to the CV 14 and reemerged to the abdominal cavity toward the liver surface. The flow line was barely visible under the stereo fluorescence microscope. Fig B in S1 Fig: Phase contrast microscope images of the boxed region in (A). The boundary of the abdominal wall fat band is indicated with two yellow curves. The PN and twisted primo vessel manifestly appear with fluorescent. The size of the PN was 250 μm. The fluorescent nanoparticles were highly concentrated in the PN. 40x. Fig C in S1 Fig: Similar to the above case the fluorescent nanoparticles flowed in a primo vessel. Fig D in S1 Fig:The PN in which the primo vessel was connected is shown. Fig E in S1 Fig: Similarly to the above two cases the flow was traced. Fig F in S1 Fig: The PN in which the FNP flowed. Fig G in S1 Fig: The primo vessel in which the FNP flowed. (TIF) [file pone.0150423.s001.tif]
